# ZnO- and TiO_2_-Based Nanostructures

**DOI:** 10.3390/nano8050325

**Published:** 2018-05-14

**Authors:** Andrea Lamberti

**Affiliations:** 1 Department of Applied Science and Technology, Politecnico di Torino, C.so Duca degli Abruzzi 24, 10129 Turin, Italy; andrea.lamberti@polito.it; 2 Center for Sustainable Future Technologies, Istituto Italiano di Tecnologia (IIT@Polito), C.so Trento 21, 10129 Turin, Italy

**Keywords:** ZnO, TiO_2_, nanostructures, smart materials

Transition-metal oxide (TMO) nanostructures are the focus of current research efforts in nanotechnology since they are the most common minerals on Earth, and also thanks to their special shapes, compositions, and chemical and physical properties. They have now been widely used in the design of energy saving and harvesting devices, such as lithium-ion batteries, fuel cells, solar cells, and even transistors, light emitting devices (LEDs), hydrogen production by water photolysis and its storage, water and air purification by degradation of organic/inorganic pollutants, bio-sensing devices, environmental monitoring by their applications in the fabrication of gas, humidity, and temperature sensors, and photodetectors. TMOs can overcome the limitation imposed by the relatively poor properties of standard electrodes, showing high carrier mobility and significantly low charge recombination rates.

In addition to the great application potentials, TMO-based nanomaterials, such as ZnO and TiO_2_, have recently revolutionized nanomaterial research thanks to their outstanding smart properties. They can be produced in different shapes (such as nanowires, nanobelts, nanorods, nanotubes, nanocombs, nanorings, nanohelixes/nanosprings, nanocages and nanosheets, and nanostars) depending on the synthesis routes, resulting in different physicochemical properties.

The present Special Issue covers the most recent advances in ZnO and TiO_2_ nanostructures, concerning not only their synthesis and characterization, but also reports of the manner(s) in which their functional and smart properties can be applied in working devices. Applications of nanosized ZnO and TiO_2_ can range widely, from biomedical and drug delivery devices to piezoelectric and chemical sensors, and energy harvesting, conversion, and storage devices.

Twenty-seven papers compose this issue, with invited contributions and regular original papers, and both reviews and research articles.

The two first reviews address the application of ZnO nanostructures to chemoresistive sensing (Rackauskas and coworkers [1], invited contribution) and Surface Enhanced Raman Scattering (by Yang et al. [2]). 

The last two reviews focus on TiO_2_ nanostructures: Wang et al. [3] describe the engineering of the surface/interface structures of titanium dioxide micro- and nano-architectures towards environmental and electrochemical applications while the wet chemical preparation of TiO_2_-based composites with different morphologies and their photocatalytic properties are reported by Xiang and Zhao [4].

Several research articles focus on the synthesis and deposition of ZnO and TiO_2_ nanostructures. Hsu et al. [5] discuss the effect of process parameters on the sputtering deposition of indium titanium zinc oxide thin film for transistor fabrication. Shih and Wu [6] investigate the growth mechanism of ZnO nanowires, providing experimental observation and providing a short-circuit diffusion analysis. The sol-gel synthesis of ZnO/ZnS heterostructures is discussed by Berbel Manaia et al. [7], focusing on the critical role of thioacetamide concentration. Chen et al. [8] report on the preparation and characterization of ZnO nanoparticles supported on amorphous SiO_2_. Rana and coworkers [9] discuss how the growth method and process parameters influence the optical, conductive, and physical properties of solution-grown ZnO nanostructures. Folger and coworkers [10] show how the heat treatments in different environments allow electronic conductivity to be tuned in hydrothermally grown rutile TiO_2_ nanowires. Jin et al. [11] present a simple and novel strategy to obtain TiO_2_ nanowire networks by titanium substrate corrosion and their application in third generation solar cells. Berthod et al. [12] show the improvement achieved in the fabrication of periodic TiO_2_ nanostructures by colloidal lithography approach. Liang and coworkers [13] describe the synthesis and characterization of organozinc precursor-derived crystalline ZnO nanoparticles. 

Some contributions discuss the photo-induced smart properties of metal-oxide nanostructures: Hu et al. [14] show how via constructing appropriate heterostructures between mesopore TiO_2_ nanospheres and Sn_3_O_4_ nanoparticles it is possible to enhanced their ultraviolet-visible light photocatalytic activity. The study of the photodynamic activity of N-doped TiO_2_ nanoparticles conjugated with aluminum phthalocyanine is reported by Pan et al. [15] Li and coworkers investigate the photoelectrochemical water splitting properties of Ti-Ni-Si-O nanostructures grown on Ti-Ni-Si alloy substrate [16]. The visible light response of mesoporous titania films loaded with silver salts is reported by Crespo-Monteiro et al. [17] for the degradation of methyl blue.

Other functional properties of ZnO and TiO_2_ nanostructures are described: the piezoelectric potential in single-crystalline ZnO nanohelices is studied by finite element analysis by Hao et al. [18] (invited contribution) while the self-cleaning behavior of a nano-TiO_2_-coated SiO_2_ microsphere composite is reported by Sun et al. [19].

The electrical properties are also analyzed: the memristive response of TiO_2_ nanoparticle is investigated by Schmidt et al. [20] while Bruzzi and coworkers [21] report the thermally stimulated current in nanocrystalline titania.

Other contributions addressed biological issues: Yamamoto et al. [22] show novel results on the in vitro sonodynamic therapeutic effect of polyion complex micelles incorporating titanium dioxide nanoparticles. Ancona et al. [23] (invited contribution) report a novel strategy for photodynamic cancer therapy exploiting lipid-coated ZnO nanoparticles. Zhang and coworkers [24] demonstrate the duplex bioactivity of titanium-based materials achieved by oxidation and nitrogen implantation. McCall et al. [25] reported the protective effect of ZnO nanoparticle for RNA, while the interaction of ZnO surface with biomatrices is discussed by Yu et al. [26].

Finally, Casu et al. [27] (invited contribution) showed how heating anodically grown TiO_2_ nanotubes in situ in a transmission electron microscope allows for the monitoring of their crystallization from amorphous to polycrystalline with polymorphs dependent on the selected temperature. 

I would like to gratefully acknowledge all the authors for their valuable contributions and expertise, as well as the reviewers for their comments and suggestions and the assistant editors for the constant support: all have contributed to the success of this special issue.

Hoping that the special issue contents provide an actual overview of the TiO_2_ and ZnO nanostructures synthesis and applications, even if not exhaustive of this huge research field, I wish you a good reading.
